# Bibliometric analysis of bacterial central nervous system infection research in Southeast Asia

**DOI:** 10.1186/s12883-021-02042-w

**Published:** 2021-01-08

**Authors:** Francis Gerwin U. Jalipa, Marie Charmaine C. Sy, Adrian I. Espiritu, Roland Dominic G. Jamora

**Affiliations:** 1 Division of Adult Neurology, Department of Neurosciences, College of Medicine – Philippine General Hospital, University of the Philippines Manila, Manila, Philippines; 2 Department of Clinical Epidemiology, College of Medicine, University of the Philippines Manila, Manila, Philippines; 3 Institute for Neurosciences, St. Luke’s Medical Center, Global City, Philippines

**Keywords:** Bacterial central nervous system infection, Bibliometric analysis, Southeast Asia

## Abstract

**Background:**

The status of research and scientific outputs with regards to bacterial central nervous system (CNS) infection in southeast Asia (SEA) is unknown. This study aimed to analyze and compare bacterial CNS infection research output of SEA countries in terms of bibliometric indices.

**Methods:**

The major electronic databases (MEDLINE, Scopus, Embase, CENTRAL, Clinicaltrials.gov and WPRIM) were searched for studies on bacterial CNS infection in SEA until August 31, 2020. Frequencies, percentages and Spearman’s rho correlations were used.

**Results:**

There was a total of 648 unique studies on bacterial CNS infection in SEA and it was 657 when double-counted (collaborative studies between SEA countries). Thailand (*n*=148, 22.5%) and Vietnam (*n*=142, 21.6%) had the highest number of publications. The most common type of research publication was the case report / case series (*n*=160, 24.7%). Tuberculous meningitis/tuberculoma (*n*=176, 26.7%) was the most common topic. This study showed that the %GDP for research and development (R&D) was associated with a higher number of research output. However, the GDP per capita was not associated with any of the bibliometric indices. The total number of neurologists was associated with all of the bibliometric analysis.

**Conclusion:**

Bacterial CNS infection research output in SEA countries was low in terms of quantity. The %GDP for R&D was associated with the number of research publications. The total number of neurologists was associated with all of the bibliometric indices.

## Introduction

Infection of the central nervous system (CNS) is one of the common neurologic conditions worldwide. CNS infections affected 10,425,058 people per year in the Southeast Asia (SEA). This remains high compared to other regions of the world [1]. There are many bacterial pathogens that are associated with invasion of the CNS. The clinical presentations of bacterial CNS infection ranges from meningitis, encephalitis, or meningoencephalitis to focal CNS syndromes such as abscess [2]. Meningitis is the most common clinical presentation. The most frequent causative agents were *Streptococcus pneumonia* and *Mycobacterium tuberculosis* [3].

Bacterial infection of the CNS presents a significant disease burden. In terms of age-adjusted disability-adjusted life-year, meningitis ranked as the 5th, encephalitis ranked as the 9th and tetanus ranked as the 13th among the 15 different neurologic conditions in the SEA region in 2016 [4]. Thus, this group of neurologic diseases, which are preventable, need to be addressed appropriately. A country’s spending on research and development (R&D), number of universities and number of indexed journals were shown to be positively correlated with the published documents, citations per documents and H-index. However, there was no correlation between the GDP per capita and research outcomes [5].

Traditionally, the research impact of an author is measured by different bibliometrics, such as journal impact factor (IF), H-index, number of publications, and number of citations [6]. Alternatively, another method of evaluating the impact of research articles can be used. This is known as alternative metrics, or altmetrics, which uses more immediate metrics. An example of this is the PlumX Metrics, which uses five major categories: Usage, Captures, Mentions, Social Media, and Citations [7].

The status of research and scientific outputs with regards to bacterial CNS infection in SEA is unknown. This is hypothesized to be low, as shown in other neurologic diseases, such as epilepsy, dementia, multiple sclerosis, Parkinson’s disease, stroke, primary brain tumor, and neuromyelitis optica spectrum disorder [8–13]. Therefore, this study aimed to determine the bacterial CNS infection research output of SEA countries in terms of bibliometric indices. The association between total publications, journals with IF, PlumX Metrics, number of neurologists per country to socioeconomic factors of a nation such as population size, GDP per capita, and %GDP for R&D were also evaluated in this study.

## Methods

A systematic review was performed to retrieve all relevant articles on bacterial CNS infection. The Preferred Reporting Items for Systematic Reviews and Meta-Analyses (PRISMA) guidelines were followed for this study [14].

### Criteria for considering studies for this review

We included studies that used any study design (randomized controlled trials, systematic reviews, meta-analysis, case-control studies, cohort studies, cross sectional studies, expert reviews, case series, case reports, animal studies and laboratory studies). We excluded studies that were conference papers, letters to the editor, book chapters, terminated studies, proceedings, commentaries, written in non-English language, and those that were done outside SEA. We considered the studies that involved animal, human or in-vitro laboratory studies in relation to bacterial CNS infection, with at least one author affiliated to any of the SEA countries [Brunei, Cambodia, Indonesia, Lao People’s Democratic Republic (Lao PDR/ Laos), Malaysia, Myanmar, Philippines, Singapore, Thailand, Timor-Leste, and Vietnam]. Studies about non-bacterial causes of CNS infection (viral, fungal, and parasitic) were excluded.

### Search methods for identification of studies and selection of studies

We searched the major electronic databases [MEDLINE by Pubmed, Scopus, Embase, Cochrane Central Register of Controlled Trials (CENTRAL), Clinicaltrials.gov and Western Pacific Regional Index Medicus (WPRIM)] for studies on bacterial CNS infection in SEA until August 2020. We used the following search terms: [(“bacterial central nervous system infection” OR “bacterial meningitis” OR “bacterial encephalitis” OR “bacterial meningoencephalitis” OR neurosyphilis OR “tuberculous meningitis” OR “central nervous system tuberculoma” OR tetanus OR “brain abscess” OR “subdural empyema” OR “subdural abscess” OR “epidural abscess” OR “spinal cord abscess”) AND (Philippines OR Indonesia OR Malaysia OR Thailand OR Cambodia OR Brunei OR Singapore OR Laos OR “Lao PDR” OR Vietnam OR Myanmar OR Burma OR Timor-Leste OR “East Timor” OR Timor)].

Two investigators (FGUJ, MCCS) were involved in the selection of studies for inclusion; if there were any disagreement, a consensus with a third investigator (AIE, RDGJ) was conducted. Predetermined screening criteria were used to screen for the titles and abstracts of the searched records. Duplicate studies were immediately discarded. The studies that fulfilled the screening criteria were obtained as full-text articles and these were reviewed for eligibility. Records that fulfilled the eligibility criteria were included in the qualitative analysis. Articles which were collaborations between/among SEA countries were double counted.

### Bibliometric indices and country specific socio-economic data

The bibliometric indices used for this study were the latest journal IF (2020) and PlumX Metrics, which organized five [5] metrics into the following broad categories: (a) Usage, which includes abstract views, figure views, PDF views, clicks, and article downloads; (b) Captures, which contains bookmarks, favorites, citation exports, and subscription to YouTube channels; (c) Mentions, which includes reviews, comments, and blog mentions; (d) Social Media, which contains the likes, tweets, and shares about the study in social media platforms; and (e) Citation, which is concerned with the traditional measures of researcher impact, such as citation indexes, clinical citations, and patent citations [7].

Information about the population size of each SEA country in 2020 was obtained from the Worldometers website [15]. Data from the International Monetary Fund website was used to attain information on the GDP per capita of each country [16]. Data from the World Bank website was used to obtain information about allocation of %GDP for research and development (R&D) in each country [17]. The data on the number of neurologists in each southeast Asian country was obtained from recent publications [8, 18, 19]. The number of neurologist-to-population ratio was then computed based on these data.

### Data analysis

Statistical analysis was performed using the SPSS version 25 (IBM Corp., Armonk, NY, USA). Frequencies and percentages were presented for the all PlumX and Scopus metrics except for the field weighted citation impact which used averages. To further explore the publications per country, graphical representations were provided using stacked bar charts depending on the category. To assess if there is a relationship between the country profiles and their citations, Spearman’s Rho correlations were done.

## Results

### Results of the systematic search

**Fig. 1:**
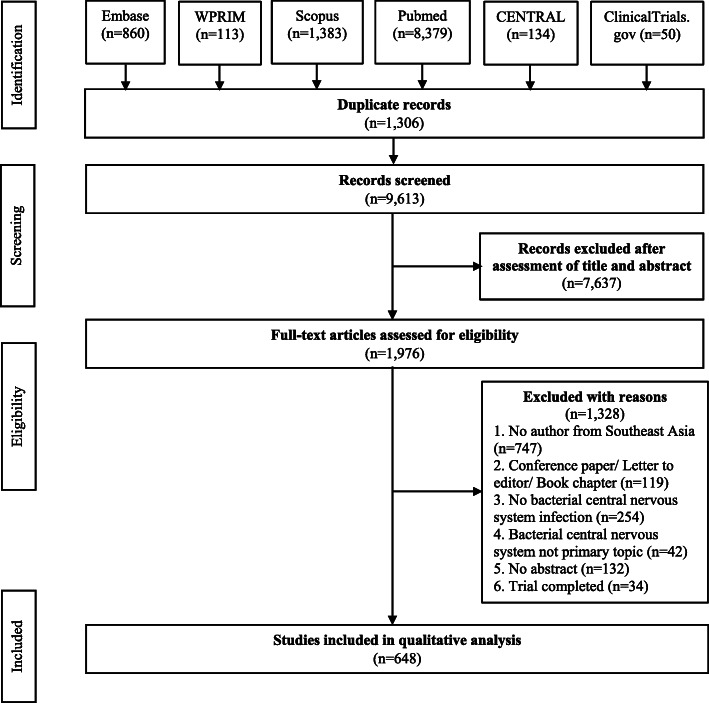
PRISMA flow diagram for study selection

A total of 10,919 articles were obtained using the search strategy. These articles were searched from the different major databases (MEDLINE: 8379; CENTRAL: 134; Scopus: 1383; Embase: 860; WPRIM: 113; Clinicaltrials.gov: 50). There were 1306 duplicate articles that were immediately excluded. Of the remaining 9613 articles, 7637 were discarded based on the screening criteria used. The full-text of 1976 articles were assessed for eligibility and 648 articles were then included for analysis (Fig. 1).

### Top journals that published SEA papers on bacterial CNS infection

A total of 648 articles published in 243 journals were included in this review. The top 16 journals, with a share of at least 1% of the total publications, are presented in Table 1. The top 3 journals were the *Southeast Asian Journal of Tropical Medicine and Public Health* (*n*=39, 6.0%), *Journal of the Medical Association of Thailand* (*n*=35, 5.4%), and *Vaccine* (*n*=30, 4.6%). It was noticeable that aside from Thailand, Singapore had its own set of journals that made it to the top 16. This included the *Annals of the Academy of Medicine Singapore* (*n*=15, 2.3%) and the *Singapore Medical Journal* (*n*=13, 2.0%). Only 6 out of these 16 journals had recent publications in 2020 (*Vaccine, Plos ONE, American Journal of Tropical Medicine and Hygiene, and Clinical Infectious Diseases, International Journal of Infectious Diseases*, and *Wellcome Open Research*). Furthermore, *Clinical Infectious Diseases* had the highest IF (8.313) with 13 articles, when compared to the other journals. The *Journal of Infectious Diseases* came next, with a 5.022 IF and 10 articles.

**Table 1:** Top 16 out of 243 Journals (at least 1% of total)

Journal	No. (*n*=648)	% of Total	2019 Impact Factor
*Southeast Asian Journal of Tropical Medicine and Public Health*	39	6.0%	0.245
*Journal of the Medical Association of Thailand*	35	5.4%	–
*Vaccine*	30	4.6%	3.143
*PLoS ONE*	20	3.1%	2.74
*Journal of Preventive Medicine*	19	2.9%	–
*Annals Academy of Medicine Singapore*	15	2.3%	1.533
*American Journal of Tropical Medicine and Hygiene*	13	2.0%	2.126
*Clinical Infectious Diseases*	13	2.0%	8.313
*Singapore Medical Journal*	13	2.0%	1.359
*BMC Infectious Diseases*	12	1.9%	2.688
*Pediatric Infectious Disease Journal*	12	1.9%	2.126
*BMJ Case Reports*	10	1.5%	–
*International Journal of Infectious Diseases*	10	1.5%	3.202
*Journal of Infectious Diseases*	10	1.5%	5.022
*Open Forum Infectious Diseases*	9	1.4%	3.656
*Wellcome Open Research*	7	1.1%	–

### Country profile and relevant bibliometrics

Table 2 shows the country-specific characteristics and number of publications for CNS infection in the SEA countries and Table 3 shows the summary of relevant bibliometrics for CNS infection in the SEA countries.

**Table 2:** Country-specific characteristics and number of publications for CNS infection in the SEA countries

Countries	Population / million	GDP / capita	% GDP for R&D	Neurologists	Number of Publications^**a**^
Brunei	0.44	64,673.30	0.04	2	1
Cambodia	16.72	4570.70	0.05	5	8
Indonesia	273.52	29,525.60	0.08	1150	60
Lao PDR	7.28	8150.80	0.04	3	9
Malaysia	32.37	12,301.80	1.26	120	111
Myanmar	54.41	5355.30	0.16	23	5
Philippines	109.58	9277.40	0.14	506	83
Singapore	5.85	101,375.80	2.19	100	90
Thailand	69.80	19,228.33	0.48	645	148
Vietnam	97.34	8374.40	0.19	800	142
Total	667.30	262,833.43	4.63	3278	657

^a^There were 648 unique articles. Articles which were collaborative between/among countries were double counted. The number of collaborative articles per country were: Laos and Thailand=2, Thailand and Vietnam=1, Malaysia and Singapore=2, Brunei and Malaysia=1, Vietnam and Indonesia=1, Cambodia and Vietnam=1, Malaysia and Thailand=1 (Total=9)

**Table 3:** Summary of relevant bibliometrics for CNS infection in the SEA countries

Countries^a^	Citations	Usage	Captures	Mentions	Social Media	Scopus Citations	Average of Scopus field weighted citation impact (FWCI)^b**,**c^	Valid Articles with Scopus (FWCI)^b^
Brunei	0	25	28	0	1	0	–	–
Cambodia	42	155	405	0	0	42	0.58	4
Indonesia	547	7537	2986	2	129	873	1.91	27
Lao PDR	75	1895	661	2	10	74	0.47	4
Malaysia	516	6636	2240	2	143	580	0.60	43
Myanmar	34	18	102	0	0	42	0.58	2
Philippines	1043	4549	3387	0	255	1346	1.70	47
Singapore	487	4330	2386	0	102	802	0.97	37
Thailand	810	14,586	4135	2	576	1644	0.70	68
Vietnam	3227	84,111	15,736	32	707	5758	2.72	81
Total [or Average for FWCI]	6781.00	123,842.00	32,066.00	40.00	1923.00	11,161.00	1.14	313.00

^a^Unless otherwise stated, metrics were from PlumX

^b^Not all articles had Scopus metrics

^c^All metrics were in terms of total except for Scopus FWCI. This value was derived from individual (non-collaborative) country studies

In terms of total publications, Thailand (*n*=148, 22.5%) and Vietnam (n=142, 21.6%) share most of the publications. Each country owns 22.53 and 21.61% of the total publications, respectively. Aside from Timor-Leste which did not have any publication, Brunei and Myanmar had the lowest number of publications -- only sharing 0.15% (n=1) and 0.76% (*n*=5) to the total publications, respectively. At least 47% of the total citations, usage, captures, and mentions came from studies in Vietnam. For social media, the top 2 was still shared by Thailand and Vietnam. Nonetheless, it was Vietnam and Indonesia who had the highest average Scopus field weighted citation impact.

When country profile and bibliometrics were merged, only the total neurologist metric was significantly associated with all bibliometric indices. Similarly, population per million was found to be positively associated with citations (PlumX and Scopus), usage, captures, social media, and Scopus field weighted citation impact. The budget for R&D (in terms of % GDP) was only associated with the number of publications. Meanwhile, GDP per capita was not found to be significantly associated with any of the bibliometrics (Table 4).

**Table 4:** Correlational analysis of country-specific characteristics and bibliometrics

Country Profile	Publication Statistics^**a**^	Spearman’s Rho Coefficient	P-value^**b**^
Population / million	Number of publications^c^	0.418	0.229
	Citations	0.745	0.013*
	Usage	0.624	0.054**
	Captures	0.685	0.029*
	Mentions	0.415	0.233
	Social media	0.578	0.080**
	Scopus citations	0.699	0.024*
	Average of Scopus field weighted citation impact (FWCI)^d,e^	0.561	0.116
	Valid articles with Scopus (FWCI)^d^	0.452	0.222
GDP per capita	Number of publications^c^	0.236	0.511
	Citations	0.103	0.777
	Usage	0.261	0.467
	Captures	0.176	0.627
	Mentions	0.020	0.956
	Social media	0.292	0.413
	Scopus Citations	0.182	0.614
	Average of Scopus field weighted citation impact (FWCI)^d,e^	0.561	0.116
	Valid articles with Scopus (FWCI)^d^	0.452	0.222
% GDP for R&D	Number of publications^c^	0.742	0.014*
	Citations	0.450	0.192
	Usage	0.468	0.172
	Captures	0.498	0.143
	Mentions	0.171	0.636
	Social media	0.494	0.147
	Scopus Citations	0.518	0.125
	Average of Scopus field weighted citation impact (FWCI)^d,e^	0.343	0.366
	Valid articles with Scopus (FWCI)^d^	0.510	0.160
Neurologists	Number of publications^c^	0.721	0.019*
	Citations	0.867	0.001*
	Usage	0.867	0.001*
	Captures	0.867	0.001*
	Mentions	0.570	0.086**
	Social media	0.778	0.008*
	Scopus citations	0.875	0.001*
	Average of Scopus field weighted citation impact (FWCI)^d,e^	0.895	0.001*
	Valid Articles with Scopus (FWCI)^d^	0.695	0.038*

^a^Unless otherwise stated, metrics were from PlumX

^b^Using Spearman’s Correlation

^c^There are 648 unique articles. Articles which were collaborative between/among countries were double counted. The number of collaborative articles per country were: Malaysia and Thailand - 4 each; Vietnam - 3; Lao PDR and Singapore - 2 each; Brunei, Cambodia, and Indonesia - 1 each

^d^Not all articles had Scopus metrics

^e^All metrics were in terms of total except for Scopus FWCI. This value was derived from individual (non-collaborative) country studies

*Significant at alpha=0.05

**Significant at alpha=0.10

### Publications by study design

**Fig. 2:**
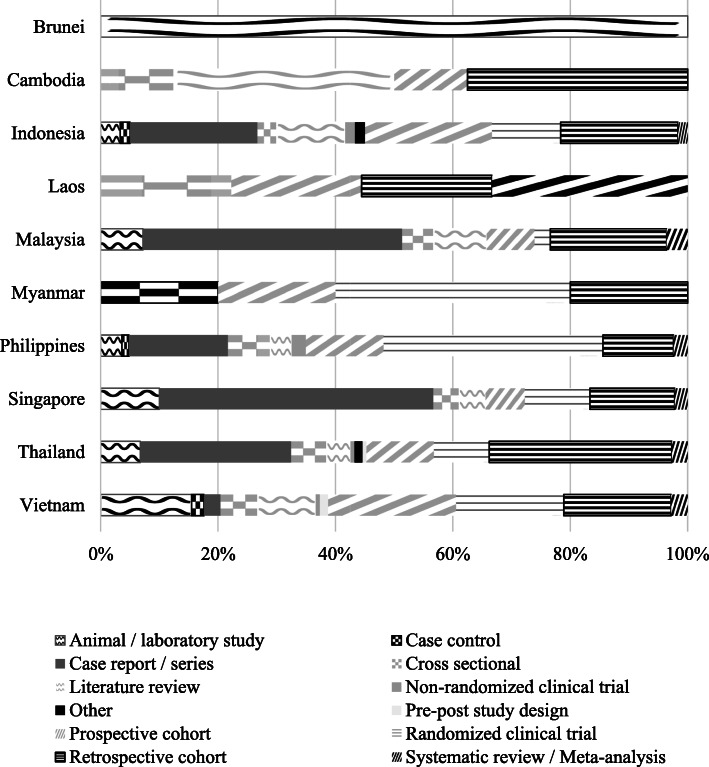
Distribution of publications (by study design) per country

The most common study designs were case report/case series (*n*=160, 24.4%), retrospective cohort (*n*=133, 20.2%), randomized clinical trial (*n*=91, 13.9%) and prospective cohort (*n*=88, 13.4%). Case-control (*n*=6), non-randomized trials (*n*=5), and pre-post study designs (*n*=3) were the least common. There were also some study designs that were unclear (tagged as “others”, n=3). In terms of country profile, the study designs were mixed. Nonetheless, it was evident that cohort designs were popular to almost all countries (Fig. 2).

### Publications by topic

Overall, the most common topics were tuberculous meningitis/tuberculoma (*n*=176, 26.8%), tetanus (*n*=148, 22.5%), gram-negative bacterial meningitis/encephalitis/meningoencephalitis (*n*=94, 14.3%), and brain or epidural or spinal cord abscess/subdural empyema (*n*=78, 11.9%). Vietnam had the highest number of publications about tuberculous meningitis/tuberculoma and tetanus while Philippines had the highest number of publications about gram-negative bacterial meningitis. The least common topics (< 5%) were neurosyphilis (n=9, 1.4%) and other spirochetal infections (*n*=2, 0.3%) (Table 5).

**Table 5:** Distribution of publications (by topic) per country

	Brunei	Cambodia	Indonesia	Laos	Malaysia	Myanmar	Philippines	Singapore	Thailand	Vietnam	Total
Brain or epidural or spinal cord abscess/subdural empyema	0	0	3	0	30	0	2	19	20	4	78
Gram-negative bacterial meningitis/encephalitis/meningoencephalitis	0	0	5	1	15	0	23	16	19	15	94
Gram-positive bacterial meningitis/ encephalitis/meningoencephalitis	1	0	3	0	7	0	15	12	17	10	65
Tuberculous meningitis/Tuberculoma	0	0	29	0	26	2	16	8	37	58	176
Mixed organism bacterial meningitis	0	3	1	2	3	2	8	5	16	11	51
Unspecified bacterial meningitis	0	3	2	2	10	1	1	7	4	4	34
Tetanus	0	2	17	3	17	0	17	19	33	40	148
Neurosyphilis	0	0	0	0	3	0	1	4	1	0	9
Other spirochetal infection	0	0	0	1	0	0	0	0	1	0	2
Total	1	8	60	9	111	5	83	90	148	142	657

**Fig. 3:**
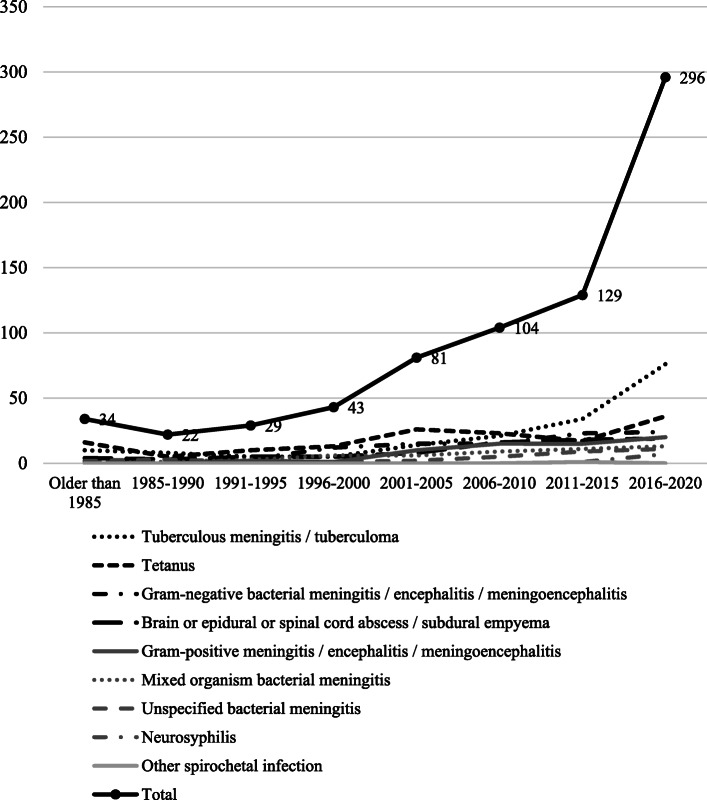
Trend in publications over the years

In general, there was a markedly small number of publications in the 1980s. However, a steadily increasing trend in the number of publications was seen over the years, with the highest numbers achieved during 2016–2020 (Fig. 3).

### Publications by research domain

**Fig. 4:**
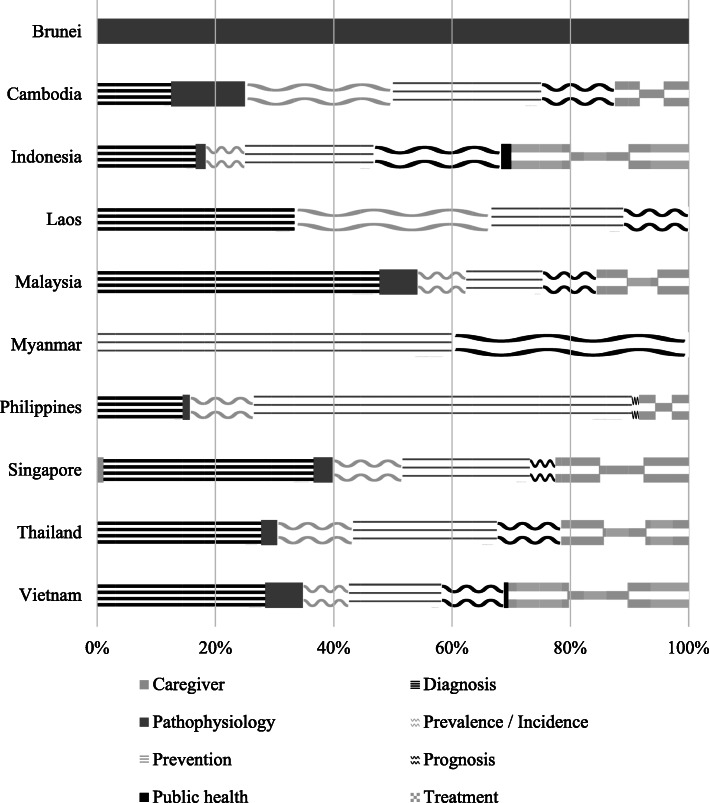
Distribution of publications (by research domain) per country

Diagnosis (*n*=192, 29.2%), prevention (*n*=165, 25.1%), and treatment (*n*=139, 21.2%) were the most common research domains. Public health and caregiving were rarely discussed (Fig. 4).

### Collaboration between SEA countries and other countries

Nine research publications involved collaborations between the following SEA countries: Laos and Thailand (*n*=2), Malaysia and Singapore (n=2), Thailand and Vietnam (*n*=1), Brunei and Malaysia (n=1), Vietnam and Indonesia (*n*=10, Cambodia and Vietnam (n=1), Malaysia and Thailand (n=1).

There was also collaboration between SEA countries and countries in different continents. The United Kingdom (*n*=117) had the highest number of collaborations between SEA countries, followed by the United States of America (*n*=69), and the Netherlands (*n*=43) (Table 6). The collaboration between Vietnam and the United Kingdom (*n*=76) topped the list, followed by Vietnam and the Netherlands (*n*=22) (Table 7).

**Table 6:** Top 5 countries with the highest number of collaborations with SEA countries

Country	Number of Publications	Citation Indexes		Citations	
		Sum	Mean	Sum	Mean
United Kingdom	117	3049	46	5517	59
United States of America	69	955	24	1694	37
Netherlands	43	1144	38	2090	61
Belgium	37	442	18	556	19
Australia	33	532	23	695	27

**Table 7:** Top collaborating countries

Country 1	Country 2	Number of Publications	Citation Indexes		Citations	
			Sum	Mean	Sum	Mean
Vietnam	United Kingdom	76	2784	56	5118	78
Vietnam	Netherlands	22	850	53	1619	90
Vietnam	United States of America	20	431	36	977	65
Philippines	Belgium	20	341	24	410	26
Philippines	United States of America	20	210	18	280	22
Philippines	Finland	18	428	25	498	29
Indonesia	Netherlands	16	265	27	438	37
Thailand	United States of America	11	98	16	145	18
Singapore	United Kingdom	10	127	32	153	22

## Discussion

This study uniquely explored the scientific impact of bacterial CNS infection among the different SEA countries. The scientific impact of the researches was assessed using the traditional bibliometric indices (total number of publications, journal citations and publication in journals with IF) supplemented by using the PlumX metrics (Usage, Capture, Mention, Social media, Citation). It correlated the socioeconomic factors of a SEA nation with research productivity.

There was a total of 648 research outputs on bacterial CNS infection in the SEA region over a period of 35 years. Among these studies, only 9 published articles had collaborations among authors from SEA. The amount of research outputs on this topic in SEA was lower compared to the other neurologic diseases (dementia, *n*=775; epilepsy, *n*=702; movement disorders, *n*=1567; stroke, *n*=2577) [9–12] but greater than primary brain tumors (*n*=549) [13]. It was notable, however, that among the published bibliometric analysis of neurology-related research outputs in SEA, studies on multiple sclerosis and neuromyelitis optica-spectrum disorders had the fewest output (*n*=142) [8]. In terms of published researches on a subtopic such as bacterial meningitis, the SEA region had fewer research outputs compared to other countries outside SEA; United States of America, *n*=2698; United Kingdom, *n* =912; Germany, *n*=749; France, *n*=620. Africa had lesser publications on this topic (less than 100) compared to SEA [20]. The bibliometric analyses of SEA research productivity on the different neurologic conditions consistently showed that Malaysia, Singapore, and Thailand produced the highest number of research publications [8–13]. In this study, Malaysia and Thailand were still among the highest producer of research outputs along with Vietnam, which replaced Singapore as part of the top 3 countries with the largest amount of publications. However, Singapore was in the 4th place. Therefore, Singapore can still be considered as a top contributor of research articles in this bibliometric analysis.

Population per million was found to be positively associated with citations (PlumX and Scopus), usage, captures, social media, and Scopus field weighted citation impact in this study. This finding could be attributed to the presence of more resources in terms of manpower, that can help promote the research content through social media, if there is a higher number of population per million in a country. These manpower resources may not necessarily need to be specialists.

The %GDP for R&D was associated with a higher total number of publications. This was seen in countries like Singapore, Malaysia, and Thailand, which had a high %GDP for R&D. This finding was consistent with the other bibliometric studies done in SEA [8, 9, 11–13]. However, %GDP for R&D was not explored in a bibliometric analysis on epilepsy research output [10]. A logical explanation can then show that if more resources are allocated for research, research productivity will expectedly increase. The GDP spent on R&D was associated with a significant impact of the scientific output in the specialty [21]. Countries that place more weight on research development and technology advancement have more research output in terms of quantity and impact [10]. However, in this study, GDP per capita was not found to be associated with any of the bibliometrics unlike the other studies. The GDP per capita was not associated with research outcomes [5].

The total number of neurologists in a country was significantly associated with all bibliometric indices. This was also seen in the other bibliometric studies [9, 10, 12]. This could be attributed to lesser patient workload and more time to perform research if there is a higher number of neurologists.

It is interesting to see that there is an increasing trend of the amount of research publications in this field. However, the numbers may be still be considered to be inadequate and more research outputs may have to be produced to address this preventable neurologic disease.

To best of our knowledge, this was the only study that measured the research productivity of bacterial CNS infection in SEA and there were no other data yet in other parts of the world with regards to this topic. The strength of this study was that it used a systematic and extensive method of searching for the relevant publications in the major electronic databases. The full-text articles were carefully screened for inclusion and analysis in this review.

This study was limited in that it only dealt with the bacterial causes of CNS infection and did not include the other etiologies such as viral, fungal and parasitic causes, which are also important causes of CNS infection [1]. Therefore, the findings of this review cannot be applied to the non-bacterial causes of CNS infection research output in SEA. Only articles from the major electronic databases (MEDLINE, Scopus, Embase, CENTRAL, WPRIM, ClinicalTrials.gov) were included in this study. We did not include the research outputs, which were difficult to retrieve such as those from non-indexed journals, local databases, proceedings, and unpublished studies. Research articles that were not written in the English language were also eliminated in this bibliometric analysis. These limitations may underestimate and may not reflect the true status and number of research outputs in SEA regarding this subject matter.

The findings of this study should not be taken negatively, but rather serve to help improve the research status of a SEA nation. It should encourage researchers to produce more research publications in the field of bacterial CNS infection as there are still many areas to be addressed. Currently, SEA has a high incidence of tuberculosis with most of the SEA nations having adequately vaccinated against this disease [21]. The mortality rate from tetanus in SEA is lower than that in Africa but is higher than Europe, Australia and the American continent [22]. For the vaccine-preventable bacterial causes of meningitis, *Haemophilus influenzae* vaccine is included in the vaccination schedule in most SEA countries while *Streptococcus pneumonia* vaccination is included in only a few countries. Since SEA is not endemic for meningococcal meningitis, *Neisseria meningitidis* vaccination is not routinely recommended [23]. A bibliometric analysis of the research outputs for the non-bacterial causes of CNS infection in SEA is recommended since there are non-bacterial etiologies that cause significant disease burden such as neurocysticercosis, viral encephalitis, and cryptococcal meningitis [1, 3]. This study can also help the administrative bodies in sound health policy formulation and allocation resources like %GDP for R&D for research in relevant CNS infections that pose a high burden to the country. Resources for manpower such as the training of more neurologists can also be considered to help increase the quality of research and address the shortage of specialists in a nation.

## Conclusion

Bacterial CNS infection research output in SEA countries was low in terms of quantity. The %GDP for R&D was associated with the number of research publications but the GDP per capita was not associated with any of the bibliometric indices. The total number of neurologists was associated with all of the bibliometric indices. This study can encourage more high-quality research publications about bacterial CNS infection and sound healthcare policy formulation.

## Data Availability

The datasets used and/or analyzed during the current study are available from the corresponding author on reasonable request.

## Abbreviations

CNS: Central nervous system
GDP: Gross domestic product
IF: Impact factor
PRISMA: Preferred Reporting Items for Systematic Reviews and Meta-Analyses
R&D: Research and development
SEA: Southeast Asia
